# Implementing Patient Decision Aids for Insulin Initiation in China: What are the Barriers and Facilitators? A TDF‐Based Qualitative Study

**DOI:** 10.1155/jdr/2842572

**Published:** 2026-07-08

**Authors:** Dan Yang, Hongzhan Jiang, Meiqi Meng, Ziyan Wang, Wanting Shen, Hongxin Liu, Haiou Yang, Xuejing Li, Yufang Hao

**Affiliations:** ^1^ School of Nursing, Beijing University of Chinese Medicine, Beijing, China, bucm.edu.cn; ^2^ Department of Nursing Administration, Fangshan Hospital Beijing University of Chinese Medicine, Beijing, China; ^3^ Beijing University of Chinese Medicine Collaborating Center of Joanna Briggs Institute, Beijing, China; ^4^ Beijing University of Chinese Medicine Best Practice Spotlight Organization, Beijing, China

**Keywords:** insulin initiation, patient decision aids, qualitative study, Theoretical Domains Framework, Type 2 diabetes mellitus

## Abstract

**Background:**

When oral antidiabetic drugs are insufficient to achieve glycemic control in Type 2 diabetes mellitus (T2DM), patients face the decision of whether to initiate insulin therapy. Patient decision aids (PtDAs) facilitate shared decision‐making (SDM) by supporting informed, preference‐congruent choices about insulin initiation. However, PtDA implementation in China remains challenging, with limited understanding of context‐specific barriers.

**Objective:**

This study is aimed at identifying the barriers and facilitators to implementing PtDAs for supporting informed decisions about insulin initiation in T2DM management in China, from the perspectives of key interest holders (patients, endocrinologists, nurses).

**Methods:**

A qualitative descriptive study was conducted using semistructured interviews guided by the Theoretical Domains Framework (TDF). A purposive sample of 26 participants (10 patients with T2DM, 6 physicians, and 10 nurses) was recruited from a university‐affiliated hospital in Beijing. Interviews were audio‐recorded, transcribed verbatim, and analyzed using a framework analysis approach assisted by NVivo.

**Results:**

Fifteen barriers and five facilitators were identified for healthcare professionals, whereas six barriers and five facilitators were identified for patients. Key barriers included a lack of knowledge about PtDAs and insufficient skills for their use among both groups. Notable barriers related to social/professional role and identity were found, with physicians often self‐identifying as sole “decision‐makers” and nurses expressing role ambiguity regarding SDM. The absence of reinforcement mechanisms and concerns about increased workload and limited resources were substantial organizational barriers. Facilitators included positive beliefs about PtDAs′ consequences and the readiness of nurses, whose established health education practices position them as potential champions for implementation. Patients demonstrated a positive intention to use PtDAs once their purpose was understood.

**Conclusions:**

The implementation of a PtDA for informed decision‐making about insulin initiation in T2DM patients in China is influenced by a complex array of factors. Moving beyond the common barrier of time constraints, this study highlights foundational gaps in knowledge, skills, and role alignment as primary hurdles. A multifaceted implementation strategy is essential, comprising comprehensive education and training, clear role delineation, adaptation of PtDAs to fit clinical workflows, and strong organizational commitment with integrated monitoring and support.

## 1. Introduction

Type 2 diabetes mellitus (T2DM) represents a formidable global health challenge, with its prevalence reaching epidemic proportions. China bears the world′s largest burden, with an estimated 140 million adults living with diabetes, a figure projected to rise further [1, 2]. Despite significant advancements in pharmacological treatments, a substantial proportion of individuals with T2DM do not achieve or maintain recommended glycemic targets (e.g., HbA1c < 7.0*%*) [3]. This suboptimal glycemic control is a primary driver of debilitating micro and macrovascular complications—including nephropathy, retinopathy, cardiovascular disease, and increased mortality—imposing a heavy economic strain on healthcare systems and diminishing patients′ quality of life [4, 5].

When glycemic control is not adequately maintained with oral antidiabetic drugs (OADs), patients and clinicians face a preference‐sensitive decision regarding treatment intensification. Options often include adding further OADs, GLP‐1 receptor agonists, or initiating various insulin regimens [6]. Each option presents unique trade‐offs regarding efficacy, side effects (e.g., hypoglycemia, weight gain), and lifestyle impact. In contemporary Chinese clinical practice, the expanding availability of newer noninsulin injectables and advanced oral agents has further diversified the therapeutic landscape, making treatment selection increasingly complex and highly dependent on individual patient values, comorbidities, and daily routines [7]. Consequently, reaching a mutually agreed‐upon management plan requires navigating substantial clinical uncertainty and aligning medical recommendations with patient priorities, a process inherently suited to shared decision‐making (SDM) [8].

SDM is a collaborative clinical process wherein clinicians and patients exchange evidence‐based information, discuss treatment preferences, and jointly arrive at healthcare decisions that reflect the best available evidence and the patient′s personal values [9]. Patient decision aids (PtDAs) are evidence‐based tools specifically designed to facilitate SDM [10]. To support this complex choice, PtDAs are designed to present the pros and cons of all available intensification strategies (e.g., insulin vs. noninsulin intensifiers) in a balanced manner. Rather than persuading patients toward a specific therapy, the PtDA aims to reduce decisional conflict by helping patients align their personal values (e.g., fear of injections vs. desire for potent glucose lowering) with the clinical evidence. Recent international consensus [11] and systematic reviews [12, 13] confirm that PtDAs significantly improve patient knowledge, calibrate risk perception, reduce decisional conflict, and enhance participation without disproportionately favoring any single intervention. It is critical to clarify that PtDAs are not behavioral nudges toward insulin initiation; rather, they are unbiased patient tools that empower patients to make informed, preference‐congruent choices across the full spectrum of available therapies.

Despite robust evidence of their efficacy, the routine integration of PtDAs into clinical workflows remains inconsistent, particularly in high‐volume healthcare systems. Successful implementation is influenced by a complex interplay of patient, provider, organizational, and systemic factors [14]. In China, contextual elements such as dense patient schedules, traditionally hierarchical consultation models, variable health literacy, and limited familiarity with structured decision‐support tools may introduce unique barriers to adoption [15]. Although several PtDAs for chronic conditions have been developed in China, systematic evaluation of their real‐world implementation determinants remains scarce [16, 17]. A comprehensive understanding of what hinders or enables PtDA integration is essential to move beyond tool development toward sustainable clinical deployment. In this study, we focus on the implementation of a PtDA designed to support informed decision‐making regarding insulin initiation, which has been developed based on existing guidelines and preliminary research, and is now ready for deployment and evaluation in clinical practice [18].

The PtDA series developed for this study comprises four paper‐based, culturally adapted booklets designed to support T2DM patients through sequential treatment intensification decisions. The four tools address distinct but interconnected decision points: Tool 1, “Should I start using insulin?” (the primary focus of this implementation study); Tool 2, “When should I start using insulin?”; Tool 3, “What are the available insulin regimens for initiation?”; and Tool 4, “What are the non‐insulin treatment options?” Each of the four tools follows a shared 6‐core‐step structure: (1) introduction to the decision context and disease background; (2) comparative analysis of the pros and cons of the available options; (3) personal values and preference clarification; (4) decisional certainty assessment; (5) identification of additional information needs; and (6) comprehensive decision review. This study focuses on Tool 1 (the insulin initiation decision), which is designed to be self‐administered or reviewed with a nurse facilitator in approximately 15–20 min. The tool is intended to serve as a conversation aid during clinical consultations, not as a substitute for professional medical advice. An example of the PtDA is provided as Supporting Information S1.

To systematically map these implementation determinants, this study employed the Theoretical Domains Framework (TDF), an integrative model synthesizing 33 behavior change theories into 14 domains encompassing knowledge, skills, social influences, environmental context, and beliefs about consequences [19]. The TDF provides a structured mechanism for identifying modifiable barriers and facilitators across different interest holders, thereby guiding the development of context‐sensitive implementation strategies [20]. This qualitative study aims to characterize the barriers and facilitators perceived by key interest holders regarding the implementation of PtDAs for insulin initiation decisions in China. The findings will provide an actionable evidence base for designing tailored strategies to support the successful integration of PtDA into routine diabetes care, ultimately improving decision quality and patient‐centered outcomes.

## 2. Materials and Methods

### 2.1. Study Design and Theoretical Framework

This study employed a qualitative descriptive design, following the approach described by Kim et al. [21], which aims to provide a comprehensive summary of participants′ experiences in everyday language, staying close to the data without high‐level abstraction. Semistructured interviews were used to explore the barriers and facilitators to implementing PtDA for informed decision‐making in T2DM in China. The reporting of this study adheres to the standards of qualitative research reporting (SRQR) guidelines [22]. This study was guided by the TDF, an integrative framework of 14 domains that synthesizes constructs from 33 theories of behavior change [19]. The TDF was selected for its comprehensive approach to identifying influences on implementation behaviors across multiple levels, including knowledge, skills, social influences, environmental context, and beliefs about consequences. This framework provided a structured lens for developing the interview guide, coding the data, and interpreting the findings.

### 2.2. Participant Recruitment and Sampling

A purposive sampling strategy was employed to recruit key interest holder groups involved in the care of patients with T2DM, ensuring a diversity of perspectives. Participants included (1) endocrinologists, (2) primary care physicians from community health centers, (3) endocrinology nurse, and (4) adult patients with T2DM who were either considering or had recently initiated insulin therapy.

Recruitment was conducted through two sites in Beijing: a university‐affiliated tertiary hospital (serving approximately 19,000 annual endocrinology outpatient visits) and a collaborating community health center. Physicians and nurses were invited via departmental meetings and secure WeChat professional groups restricted to staff affiliated with these two institutions. Patients were identified and screened by endocrinology nurses, with study information disseminated through diabetes management WeChat groups and posters in the clinic. Eligible participants were provided with detailed study information and invited to participate. All recruited patients were receiving care at either the tertiary hospital or the community center, ensuring that all participants were embedded within the same regional healthcare network.

The inclusion criteria for medical staff were (1) registered physician or nurse; (2) ≥ 5 year of clinical experience in T2DM management; (3) direct involvement in prescribing, adjusting, or educating patients on insulin therapy; and (4) voluntarily participate and grant informed consent. The exclusion criteria for medical staff were (1) having no direct responsibility for T2DM patient management and (2) being on extended leave (e.g., maternity, sick, or study leave) during the study recruitment period.

The inclusion criteria for patients were (1) diagnosis of Type 2 diabetes; (2) being ≥ 18 years old; (3) suboptimal glycemic control with oral hypoglycemic agents and who have been advised by doctors to start using insulin; (4) ability to communicate in Mandarin; and (5) voluntarily participate and grant informed consent. The exclusion criteria for patients were (1) severe cognitive impairment that would preclude informed consent or meaningful interview participation; (2) diagnosed with Type 1 diabetes or gestational diabetes; (3) currently participating in another interventional study that might influence their views on diabetes treatment; and (4) severe uncorrected hearing or speech impairment that prevents verbal communication.

The sample size for the interviews was determined based on the principle of data saturation. Data saturation was assessed separately for the patient group and the healthcare professional group. According to the concept of “information power” proposed by Malterud et al. [23], a medium‐sized sample is generally sufficient to obtain rich information when the study has a clear theoretical framework, employs in‐depth interviews, and includes diverse populations. Vasileiou et al. [24], through a systematic analysis, found that the median effective sample size for qualitative interview studies in the health field is approximately 25 participants. This study recruited 10 patients and 16 healthcare professionals. During the research process, the investigators continuously analyzed the data. Patient saturation was reached after the 8th interview, with 2 additional interviews confirming no new themes. Professional saturation was achieved after the 14th interview, with subsequent interviews yielding no novel codes.

### 2.3. Data Collection

Semistructured interviews were conducted between January 2025 and June 2025. A semistructured interview guide comprising 50 core questions (25 for patients and 25 for medical staff) with domain‐specific probes was developed based on the 14 domains of the TDF to ensure all relevant theoretical constructs were explored (see Supporting Information S2). The outline was refined after pilot testing with one endocrinologist, one nurse, and one patient to improve clarity and flow.

All interviews were conducted one‐on‐one and face‐to‐face in a private room at the hospital or via a secure teleconferencing platform, based on participant preference. Each interview lasted approximately 40–60 min. Prior to interviews, participants were provided with the PtDA and allotted 15 min to review its content. None reported prior clinical exposure to PtDA. Prior to the interview, written informed consent was obtained from all participants. With permission, all interviews were audio‐recorded and transcribed verbatim by a professional transcription service. The transcripts were subsequently deidentified and checked for accuracy against the audio recordings by the research team.

### 2.4. Data Analysis

The interview data were analyzed using the framework analysis method [25], a systematic and flexible qualitative approach well‐suited for applied policy research and studies that have specific research questions and utilize a preexisting theoretical framework (the TDF). The analysis was conducted in five key stages, with the process managed using NVivo 14.0 software to facilitate data organization and retrieval.

In terms of familiarization, the research team (D.Y. and H.J.) immersed themselves in the data by repeatedly reading the interview transcripts to gain a comprehensive overview of the content and context.

In terms of identifying a thematic framework (coding), an initial coding framework was developed deductively based on the 14 domains of the TDF. Two researchers (D.Y. and H.J.) independently applied this framework to code the first three transcripts. They employed a combined deductive and inductive strategy. Data were primarily categorized into the relevant TDF domain (deductive) while allowing new context‐specific codes to emerge from the data that did not neatly fit into the predefined domains (inductive) [26]. These emergent codes were discussed and assigned to the most relevant TDF domain or noted for further review. The researchers then met to compare their coding, resolve discrepancies through discussion, and refine the code definitions and the framework itself. This process ensured consistency and rigor, resulting in a robust, consensus‐based coding framework.

In terms of indexing (applying the framework), the refined thematic framework was systematically applied to the entire dataset. One researcher (D.Y.) coded all remaining transcripts using the finalized codebook in NVivo.

In terms of charting (data summarization and synthesis), to facilitate a domain‐based analysis, a series of thematic charts (matrices) were created within Microsoft Excel, with each row representing a participant and each column representing a TDF domain. Key coded data and illustrative quotes from each transcript were summarized and synthesized into the corresponding cells of these matrices. This process organized the fragmented coded data into a structured format, allowing for a clear overview of the barriers and facilitators within and across each TDF domain for both patient and healthcare professional groups.

In terms of mapping and interpretation, the completed charts were used to identify patterns, associations, and key themes. The research team examined the data within each TDF domain to define the nature of the barriers and facilitators. It is important to note that the primary analysis and determination of data saturation were conducted for two macro‐groups: patients and healthcare professionals. Subsequently, for interpretative purposes, the healthcare professional group was further disaggregated into physicians and nurses to explore role‐specific nuances, and the findings were compared and contrasted across these subgroups to draw conclusions about the primary influences on PtDA implementation.

To ensure the credibility of the analysis, all transcripts were primarily coded by one researcher (D.Y.), whereas the second researcher (H.J.) independently reviewed the coding of a subset of the transcripts and the resulting thematic charts. Any disagreements in interpretation were resolved through team discussions until a consensus was reached. Furthermore, the continuous comparison of data and the pursuit of data saturation enhanced the consistency and depth of the analysis.

During translation process, all interviews were conducted in Mandarin Chinese. Verbatim transcripts were translated into English by a bilingual research assistant. The second researcher (H.J.) back‐translated a random 20% of the quotes to verify accuracy. Any discrepancies were resolved by consensus among the research team (D.Y., H.J., and X.L.).

All interviews were conducted and transcribed in Mandarin Chinese. The primary coding and framework analysis were performed on the original Mandarin transcripts. Selected illustrative quotes were later translated into English for publication.

### 2.5. Reflexivity and Trustworthiness

To ensure the rigor and trustworthiness of this qualitative study, we adhered to Guba and Lincoln′s [27] criteria for credibility, dependability, confirmability, and transferability. Reflexivity was systematically maintained throughout the research process to address researcher positionality and potential biases. The primary interviewer and coder (D.Y.) and the second coder (H.J.) are both PhD candidates in Nursing with extensive clinical experience in endocrinology and diabetes care. This shared professional background provided them with a deep understanding of the clinical context, including the complexities of insulin initiation and the hierarchical dynamics of Chinese healthcare settings. However, we recognized that their clinical “insider” status might predispose them to interpret data through a traditional medical lens or empathize excessively with workload constraints. To mitigate this, we engaged in bracketing by maintaining reflective journals before, during, and after data collection. These journals documented our preconceptions about SDM barriers (e.g., assumptions about physician resistance or patient health literacy) and allowed us to consciously set aside these biases during coding and interpretation.

Furthermore, trustworthiness was established through several strategies:

For credibility, we employed investigator triangulation, where D.Y. and H.J. independently coded a subset of transcripts and resolved discrepancies through consensus. Peer debriefing sessions were held with senior supervisors (X.L. and Y.H.), who are experienced qualitative researchers but not directly involved in daily clinical care to challenge interpretations and ensure they were grounded in the data.

When it comes to dependability, an audit trail was maintained, documenting all decisions made during data collection, coding, and theme development.

Regarding confirmability, to ensure findings reflected participant voices rather than researcher bias, we used direct verbatim quotes to support each theme.

When it comes to transferability, we provided thick descriptions of the study context (including hospital size and community center roles), participant characteristics, and the specific PtDA intervention, allowing readers to assess the applicability of findings to other similar healthcare settings.

### 2.6. Ethical Considerations

This study was approved by the Institutional Review Board of Beijing University of Chinese Medicine (Approval Number: 2024BZYLL0709). All procedures performed in studies involving human participants were in accordance with the ethical standards of the institutional and national research committee and with the 1964 Helsinki declaration and its later amendments or comparable ethical standards. Written informed consent was obtained from all individual participants included in the study. Participants were informed that their participation was voluntary and that they could withdraw at any time without any consequences to their medical care. All data were anonymized to protect participant confidentiality.

## 3. Results

### 3.1. Participant Characteristics

In accordance with the principle of data saturation, a total of 26 participants were included in this study, comprising 10 patients with T2DM and 16 healthcare professionals (6 physicians and 10 nurses). During data collection, the research team employed a continuous comparative analysis approach, monitoring thematic saturation separately for the patient group and the healthcare professional group. The patient group reached preliminary saturation after the 8th interview, following which 2 additional patients were recruited for verification. The healthcare professional group was analyzed as a whole, reaching thematic saturation after the 14th interview (following the 5th physician and 8th nurse), with the subsequent 2 interviews yielding no new coding categories or conceptual insights.

#### 3.1.1. Characteristics of Healthcare Professionals

The study interviewed 16 healthcare professionals, including 6 physicians and 10 nurses. The majority were female (14, 87.50%). Ages ranged from 27 to 52 years. Educational backgrounds included two (12.50%) with doctoral degrees, five (31.25%) with master′s degrees, and nine (56.25%) with bachelor′s degrees. Their work experience in the field of diabetes ranged from 6 to 14 years. Professional titles included 2 (12.50%) senior titles, 10 (62.50%) intermediate titles, and 4 (25.00%) junior titles. None had prior clinical exposure to PtDA. Detailed characteristics are presented in Table 1.

**Table 1:** Characteristics of healthcare professionals (*n* = 16).

ID	Age	Sex	Education	Professional title	Working years	Prior PtDA experience
D1	34	Female	Master	Intermediate	8	None
D2	42	Female	Master	Intermediate	7	None
D3	37	Female	Master	Intermediate	6	None
D4	48	Female	PhD	Senior	10	None
D5	52	Male	PhD	Senior	13	None
D6	42	Male	Master	Intermediate	10	None
N1	27	Female	Bachelor	Junior	6	None
N2	29	Female	Bachelor	Junior	5	None
N3	35	Female	Master	Intermediate	11	None
N4	34	Female	Bachelor	Intermediate	10	None
N5	45	Female	Bachelor	Intermediate	14	None
N6	50	Female	Bachelor	Intermediate	12	None
N7	27	Female	Bachelor	Junior	5	None
N8	28	Female	Bachelor	Junior	5	None
N9	32	Female	Bachelor	Intermediate	7	None
N10	33	Female	Bachelor	Intermediate	7	None

Abbreviations: D, physician; N, nurse; PtDA, patient decision aid.

#### 3.1.2. Characteristics of Patients

Ten patients with T2DM were interviewed, with an equal sex distribution (5 males, 50%; 5 females, 50%). Ages ranged from 41 to 65 years. Educational levels included junior high school (2, 20%), technical secondary school (1, 10%), high school (4, 40%), college (2, 20%), and undergraduate (1, 10%). None had prior exposure to PtDA. Basic patient characteristics are detailed in Table 2.

**Table 2:** Characteristics of patient participants (*n* = 10).

ID	Age	Sex	Insulin initiated	Disease duration (years)	Education level	Prior PtDA experience
P1	43	Female	Yes	5	College	None
P2	41	Female	Yes	6	Undergraduate	None
P3	50	Female	No	2	High School	None
P4	52	Female	Yes	7	High School	None
P5	55	Female	Yes	10	High School	None
P6	60	Male	No	4	Junior High	None
P7	42	Male	No	2	College	None
P8	57	Male	No	3	High School	None
P9	65	Male	Yes	14	Technical secondary school	None
P10	63	Male	Yes	12	Junior High	None

Abbreviation: PtDA, patient decision aid.

### 3.2. Barriers and Facilitators to PtDA Implementation

Through the analysis and synthesis of the interview data, barriers and facilitators were identified across the 14 theoretical domains of the TDF. For the healthcare professionals, 15 barriers and 5 facilitators were identified across domains including knowledge, skills, social/professional role and identity, beliefs about capabilities, goals, environmental context and resources, social influences, and others. For the patients, 6 barriers and 5 facilitators were identified across domains such as knowledge, beliefs about consequences, goals, environmental context and resources, and intentions. The specific factors are summarized in Table 3.

**Table 3:** Barriers and facilitators to PtDA implementation (*n* = 26).

TDF domain	Patient level	Healthcare professional level
Knowledge	※Lack of awareness and understanding of PtDAs	※ Insufficient knowledge of PtDA content
Skills	※ Lack of skills to use PtDAs	※ Insufficient skills to guide PtDA implementation ★ Existing foundation in health education practices
Social/professional role and identity	★ Acceptance of patient′s role in decision‐making	※ Physicians self‐identify as “decision‐makers” not “guides” ※ Role ambiguity and scope‐of‐practice constraints ★Alignment with professional values and evolving role identity
Beliefs about capabilities	※ Lack of confidence in using PtDAs for decision‐making	※ Lack of confidence in using PtDAs ★ Belief that PtDAs can enhance decision‐guiding capabilities
Reinforcement	—	※ Lack of performance evaluation and incentive mechanisms
Intentions	★ Positive intention to use PtDAs during medical consultations	※ Lack of motivation to provide decision support following PtDAs
Beliefs about consequences	★Belief in enhanced understanding and decisional clarity	★ Recognition of PtDAs′ positive impact on clinical decision‐making
Optimism	★Positive attitude toward PtDAs for assisted decision‐making education	★ Optimism about the gradual clinical implementation of PtDAs ※ Concern that PtDAs may increase decision complexity
Environmental context and resources	※ Limited PtDA formats, lack of diverse presentation methods	※ Inadequate PtDA support resources, lack of diverse formats ※ Discrepancy between PtDA options and actual clinical decision options ※ Human resource constraints and increased workload
Memory, attention, and decision processes	※Habituated to doctor‐led decisions, lack of participation awareness ★Willingness to actively participate, seeking informational autonomy	※ Lack of habit in integrating PtDAs into clinical decision processes
Social influences	※ Low awareness of PtDAs, lack of public promotion	※ Insufficient organizational culture ※ Limited leadership support
Behavioral regulation	—	※ Lack of monitoring mechanisms for decision‐making behaviors

*Note:* The symbol “※” denotes barrier. The symbol “★” denotes facilitator.

Abbreviation: PtDA, patient decision aid.

#### 3.2.1. Knowledge

##### 3.2.1.1. Patient Level

###### 3.2.1.1.1. Lack of Awareness and Knowledge Regarding PtDAs

Most patients lacked basic understanding of the concept, function, and usage of PtDA. Patients were often unclear about how PtDA could help them make decisions aligned with their personal values and how to effectively utilize these tools. This knowledge gap extended beyond unfamiliarity with the tool itself to include misunderstandings about its role and value in patient‐provider communication. Some patients expressed that medical decisions should be entirely the doctor′s responsibility, not understanding the patient‐participatory nature of PtDA (see Supporting Information S3 for full quotes).

##### 3.2.1.2. Healthcare Professional Level

###### 3.2.1.2.1. Insufficient Knowledge of PtDA Content

Although some healthcare professionals were aware of the basic concept of PtDAs, substantial gaps existed in their specific content knowledge. Many reported lacking a deep understanding of the design principles, evidence base, and content of PtDAs. Furthermore, limited knowledge about the sources of PtDA content and how to assess PtDA quality affected their trust in the tools and their enthusiasm for promoting them in clinical practice (see Supporting Information S3 for full quotes).

#### 3.2.2. Skills

##### 3.2.2.1. Patient Level

###### 3.2.2.1.1. Lack of Skills to Use PtDAs

Most patients lacked the practical skills to effectively use PtDAs, particularly in understanding data presentation, preference clarification, and comparing different options. Patients often struggled to use PtDAs independently and had difficulty translating the information into personal decision‐making rationale. Patients with lower education levels found usage particularly challenging and exhibited substantial discomfort when confronted with PtDAs (see Supporting Information S3 for full quotes).

##### 3.2.2.2. Healthcare Professional Level

###### 3.2.2.2.1. Insufficient Skills to Guide PtDA Implementation

Despite their professional medical knowledge, healthcare professionals demonstrated considerable shortcomings in the skills required to guide patients in using PtDAs and integrating the tools into routine clinical workflows. Many were uncertain about how to efficiently guide patients through PtDAs within limited consultation times and were unfamiliar with handling patient questions and difficulties that arose during use (see Supporting Information S3 for full quotes).

###### 3.2.2.2.2. Existing Foundation in Health Education Practices

Despite the skill gaps, the existing health education habits of healthcare professionals were identified as an important facilitator for PtDA implementation. Most professionals were already accustomed to explaining knowledge about insulin, oral hypoglycemic drugs, and traditional Chinese medicine therapies to patients, providing a solid foundation for PtDA adoption (see Supporting Information S3 for full quotes).

#### 3.2.3. Social/Professional Role and Identity

##### 3.2.3.1. Patient Level

###### 3.2.3.1.1. Acceptance of Patient′s Role in Decision‐Making

Some patients recognized and accepted their active participatory role in insulin initiation decisions, providing a basis for PtDA implementation (see Supporting Information S3 for full quotes).

##### 3.2.3.2. Healthcare Professional Level

###### 3.2.3.2.1. Physicians Self‐Identify as “Decision‐Makers” Not “Guides”

Some healthcare professionals′ professional role identity remained rooted in the traditional “decision‐maker” model, conflicting with the “decision guide” role promoted by PtDAs, potentially hindering their use (see Supporting Information S3 for full quotes).

• Role ambiguity and scope‐of‐practice constraints. Nurses expressed substantial ambiguity regarding their role in SDM for insulin initiation, primarily driven by structural constraints in their scope of practice. Unlike physicians, who hold prescribing authority, nurses identified themselves mainly as executors of medical orders and providers of health education. This professional boundary created uncertainty about their mandate to facilitate treatment decisions, particularly those involving the initiation of new therapies like insulin (see Supporting Information S3 for full quotes).
• Alignment with professional values and evolving role identity Despite the structural and identity barriers described above, some healthcare professionals recognized PtDA implementation as consistent with their evolving professional values and goals. This recognition manifested in two distinct forms: instrumental utility and normative duty (see Supporting Information S3 for full quotes).

#### 3.2.4. Beliefs About Capabilities

##### 3.2.4.1. Patient Level

###### 3.2.4.1.1. Lack of Confidence in Using PtDAs for Decision‐Making

Most patients lacked confidence in their ability to understand and use PtDAs for decision participation. Some questioned their capacity to comprehend professional medical information and expressed concerns about making wise judgments when faced with complex integrated traditional Chinese and Western medicine treatment options. Additionally, some patients had psychological reservations about assuming responsibility for treatment decisions (see Supporting Information S3 for full quotes).

##### 3.2.4.2. Healthcare Professional Level

###### 3.2.4.2.1. Lack of Confidence in Using PtDAs

Some healthcare professionals expressed doubts about their own ability to use PtDAs to guide patient decision‐making. This lack of belief in their capability potentially affected their enthusiasm for promoting and applying the tool (see Supporting Information S3 for full quotes).

###### 3.2.4.2.2. Belief That PtDAs Can Enhance Decision‐Guiding Capabilities

Some healthcare professionals held positive capability beliefs, perceiving that PtDAs could enhance their decision‐guiding abilities (see Supporting Information S3 for full quotes).

#### 3.2.5. Reinforcement

##### 3.2.5.1. Healthcare Professional Level

###### 3.2.5.1.1. Lack of Performance Evaluation and Incentive Mechanisms

The widespread lack of mechanisms incorporating patient participation in decision‐making into the performance evaluation and incentive systems for healthcare professionals was a major barrier to effective PtDA implementation. Professionals noted that using PtDAs to promote patient participation was not explicitly recognized or rewarded within the existing performance appraisal system, leading to a lack of motivation and support for their use amidst busy clinical work (see Supporting Information S3 for full quotes).

#### 3.2.6. Intentions

##### 3.2.6.1. Patient Level

###### 3.2.6.1.1. Positive Intention to Use PtDAs During Medical Consultations

In contrast to the hesitation among some professionals, most patients demonstrated positive intentions toward using PtDAs for participation in insulin initiation decisions. This positive attitude stemmed from patients′ desire for more disease information, willingness to participate in their own health decisions, and interest in integrated traditional Chinese and Western medicine intervention options (see Supporting Information S3 for full quotes).

##### 3.2.6.2. Healthcare Professional Level

###### 3.2.6.2.1. Lack of Motivation to Provide Decision Support Following PtDAs

Although some professionals acknowledged the value of PtDAs, time pressures in clinical work and the learning investment required to master the new tool reduced their willingness to use them. Professionals commonly reported that in busy clinical settings, using PtDAs might be perceived as an additional burden rather than an integral part of routine work (see Supporting Information S3 for full quotes).

#### 3.2.7. Beliefs About Consequences

##### 3.2.7.1. Patient Level

###### 3.2.7.1.1. Belief in Enhanced Understanding and Decisional Clarity

Patients generally held positive beliefs about the consequences of using PtDAs, primarily emphasizing their role in clarifying complex medical information and reducing decisional uncertainty. Rather than viewing the tool as a direct predictor of health outcomes, patients valued its ability to bridge knowledge gaps (see Supporting Information S3 for full quotes).

##### 3.2.7.2. Healthcare Professional Level

###### 3.2.7.2.1. Recognition of PtDAs′ Positive Impact on Clinical Decision‐Making

Despite practical barriers, the vast majority of healthcare professionals acknowledged the potential positive value of PtDAs. This belief in positive consequences was mainly reflected in improved patient adherence, increased treatment satisfaction, and reduced subsequent communication costs, forming an important motivation for professionals willing to try PtDAs (see Supporting Information S3 for full quotes).

#### 3.2.8. Optimism

##### 3.2.8.1. Patient Level

###### 3.2.8.1.1. Positive Attitude Toward PtDAs for Assisted Decision‐Making

Most patients showed obvious optimism toward the introduction of PtDAs for insulin initiation as decision‐support tools in hospitals. They believed such tools could improve their understanding of the disease and treatment options, enhance communication with doctors, and ultimately help them choose more suitable plans (see Supporting Information S3 for full quotes).

##### 3.2.8.2. Healthcare Professional Level

###### 3.2.8.2.1. Optimism About the Gradual Clinical Implementation of PtDAs

Healthcare professionals generally expressed optimism about the clinical implementation of PtDAs, particularly regarding a gradual rollout approach. They believed PtDAs could improve patient education quality, standardize clinical decision‐making processes, promote patient‐provider communication, and potentially enhance treatment adherence and outcomes for T2DM patients (see Supporting Information S3 for full quotes).

###### 3.2.8.2.2. Concern That PtDAs May Increase Decision Complexity

Despite general optimism, some professionals also expressed concerns. They worried that PtDAs might increase the complexity of the decision‐making process, particularly for patients with limited cognitive abilities or lower education levels, who might feel confused by information overload, potentially affecting the efficiency and quality of medical decisions (see Supporting Information S3 for full quotes).

#### 3.2.9. Environmental Context and Resources

##### 3.2.9.1. Patient Level

###### 3.2.9.1.1. Limited PtDAs Formats and Lack of Diverse Presentation Methods

The current PtDAs for insulin initiation primarily existed in a single format (mainly paper‐based materials combined with some videos), potentially failing to meet the needs of diverse patient groups. Patients expressed a need for richer, more accessible PtDA formats, such as digital versions, video explanations, and mobile applications. This limited format restricted the accessibility and convenience of PtDAs, especially for younger patients, those with visual impairments, or those preferring digital tools (see Supporting Information S3 for full quotes).

##### 3.2.9.2. Healthcare Professional Level

###### 3.2.9.2.1. Inadequate PtDAs Support Resources and Lack of Diverse Formats

Healthcare professionals similarly pointed out the issue of insufficient PtDAs support resources. They believed that to meet the needs of different patient groups, PtDAs should be provided in multiple formats, including paper versions, digital versions, video explanations, and mobile applications. Diverse formats could not only increase patient acceptance but also enhance the effectiveness of information delivery, but current medical institutions lacked the capacity to develop and maintain these varied resources (see Supporting Information S3 for full quotes).

###### 3.2.9.2.2. Discrepancy Between PtDAs Options and Actual Clinical Options

Although PtDAs were developed based on clinical guidelines, there was a gap between the intervention options presented to patients and actual clinical practice. This mismatch could lead to unrealistic patient expectations or situations where patients encountered inconsistencies between the PtDA descriptions and actual treatment processes, thereby affecting the effectiveness of communication and decision‐making (see Supporting Information S3 for full quotes).

###### 3.2.9.2.3. Human Resource Constraints and Increased Workload

Healthcare professionals generally worry that the implementation of PtDA would add to their already heavy workloads. Given the current tight medical resources and high workload, introducing PtDAs might require additional time to explain tool usage, answer patient questions arising from increased information exposure, and manage the increased communication complexity due to greater decision participation. This potential increase in workload and the associated adjustments to workflow, without corresponding support measures, could reduce professionals′ acceptance and enthusiasm for implementation (see Supporting Information S3 for full quotes).

#### 3.2.10. Memory, Attention, and Decision Processes

##### 3.2.10.1. Patient Level

• Habituated to doctor‐led decisions and lack of participation awareness. Some patients were accustomed to the traditional “doctor‐led” decision‐making model and tended to completely hand over medical decision‐making power to doctors, lacking the awareness and habit of actively participating in decision‐making. This deeply ingrained perception of a passive role reduces the demand for and acceptance of PtDAs, which may lead them to think that using PtDAs is unnecessary, and even question why doctors do not directly tell them what to do (see Supporting Information S3 for full quotes).
• Willingness to actively participate and seeking informational autonomy. Another segment of patients, particularly those with higher education levels or younger age, demonstrated a strong willingness to participate in medical decisions. These patients desired more information about T2DM knowledge and options at the insulin initiation stage, valued their own proactivity in the decision process, and believed PtDAs could help them make decisions more aligned with their personal values and preferences (see Supporting Information S3 for full quotes).

##### 3.2.10.2. Healthcare Professional Level

###### 3.2.10.2.1. Lack of Habit in Integrating PtDAs Into Clinical Decision Processes

Among healthcare professionals, the study found a general lack of habit in integrating PtDAs into daily clinical decision‐making processes. Long accustomed to making treatment decisions based on their own professional knowledge and experience and directly conveying these decisions to patients, introducing PtDAs meant changing existing work patterns. This change in habitual behavior requires time and continuous conscious adjustment (see Supporting Information S3 for full quotes).

#### 3.2.11. Social Influences

##### 3.2.11.1. Patient Level

###### 3.2.11.1.1. Low Awareness of PtDAs and Lack of Public Promotion

Most patients lacked basic understanding of PtDAs prior to exposure, severely impacting their acceptance and willingness to participate. Due to the lack of broad social publicity and popularization education, patients often felt confused, even skeptical and resistant, about the purpose, value, and usage of PtDAs (see Supporting Information S3 for full quotes).

##### 3.2.11.2. Healthcare Professional Level

###### 3.2.11.2.1. Insufficient Organizational Culture

Healthcare institutions generally lacked an organizational culture that encouraged and supported patient participation in medical decisions. The traditional medical model emphasized physician authority and professional judgment, paying less attention to patient values and preferences. This organizational culture created resistance to the introduction of PtDAs, as it potentially challenged existing decision‐making modes and power structures (see Supporting Information S3 for full quotes).

• Limited leadership support. The level of support from institutional leadership directly impacted the effectiveness of PtDA implementation. When leadership lacked clear supportive attitudes, resource allocation, and policy guarantees, frontline healthcare professionals often lacked the motivation and confidence to use PtDAs (see Supporting Information S3 for full quotes).

#### 3.2.12. Behavioral Regulation

##### 3.2.12.1. Healthcare Professional Level

###### 3.2.12.1.1. Lack of Monitoring Mechanisms for Decision‐Making Behaviors

This domain concerns how individuals and organizations monitor, plan, and change behaviors to achieve specific goals. Healthcare professionals generally reported that in the absence of clear monitoring mechanisms, the use of PtDAs might become perfunctory or gradually fade away. Furthermore, the lack of feedback mechanisms promoting changes in decision‐making behaviors made it difficult for professionals to assess whether their practice aligned with PtDA principles and requirements, hindering continuous improvement (see Supporting Information S3 for full quotes).

## 4. Discussion

This study provides a comprehensive, theory‐informed analysis of the barriers and facilitators influencing the implementation of PtDAs. Specifically, it examines informed decision‐making about insulin initiation among patients with T2DM and healthcare professionals in China. The results identified 15 barriers at the healthcare professional level and 6 barriers at the patient level, along with 5 facilitators for each group. The findings offer crucial insights for developing targeted strategies to promote the successful integration of PtDAs into routine diabetes care in the Chinese context.

### 4.1. Key Barriers: Cognitive Gaps and Structural Role Constraints

Unlike implementation studies in Western settings where consultation duration is frequently cited as the principal barrier [28, 29], our data indicate that limited familiarity with PtDA architecture and insufficient guidance competencies constitute more pressing challenges. This divergence likely reflects the nascent stage of PtDA dissemination in China, where conceptual awareness of SDM has grown but hands‐on experience with evidence‐based decision‐support tools remains limited [16, 30]. Professionals reported uncertainty regarding PtDA validation metrics and practical facilitation techniques, underscoring the need for competency‐based training rather than passive tool distribution.

Professional role identity emerged as a critical structural barrier. Many physicians maintained a traditional decision‐maker orientation, contrasting with the facilitative role mandated by SDM frameworks [9, 31]. This aligns with documented hierarchical consultation patterns in Chinese healthcare settings [15, 31]. Nurses, meanwhile, expressed role ambiguity, frequently delimiting their responsibilities to health education rather than decision facilitation, constrained by prescribing authority and institutional role definitions. Our reframed theme, “Role Ambiguity and Scope‐of‐Practice Constraints,” clarifies that this hesitation stems not from a lack of professional responsibility, but from legitimate boundaries within the current division of clinical labor. The absence of organizational reinforcement, such as performance metrics or workload credits for SDM activities, further sustains traditional practice patterns, rendering PtDA use discretionary rather than standard care [32]. Importantly, as emphasized by recent SDM consensus statements, PtDAs are unbiased patient tools designed to support preference‐congruent choices across all guideline‐concordant options (e.g., oral agents, GLP‐1 RAs, insulin), rather than behavioral nudges toward any single therapy [11, 13].

### 4.2. Facilitators: Enhanced Decisional Clarity and the Strategic Role of Nurses

Despite the barriers, several strong facilitators provide a foundation for intervention. Both patients and healthcare professionals held positive beliefs about the consequences of PtDAs, anticipating improvements in communication, patient understanding, and long‐term adherence to the treatment plan that patients and clinicians jointly select through the SDM process. This optimism, particularly regarding the potential for PtDAs to reduce future communication burdens, is a powerful motivator that can be leveraged during implementation [33].

Notably, the nursing population demonstrates the potential to serve as “champions” for the implementation of PtDAs. The findings indicate that nurses hold more positive attitudes toward PtDAs compared with physicians. They are more inclined to believe that PtDAs can effectively encourage patients to express their preferences and are more willing to actively utilize PtDAs in clinical practice. This finding is consistent with the research by Joseph‐Williams et al. [34]. The established foundation of health education practices among nurses aligns well with the content of PtDAs, positioning them as ideal candidates for introducing and guiding patients in the use of PtDAs [13]. By empowering nurses to take the lead in the initial introduction and explanation of PtDAs, the time pressure on physicians during outpatient consultations can be effectively alleviated while fully leveraging the unique advantages of nurses in patient education and communication. Additionally, a segment of patients, particularly those who were younger or more educated, expressed a strong desire for active participation and informational autonomy. This growing patient readiness for SDM should be nurtured through public awareness and clinic‐level encouragement [35].

### 4.3. Clarifying Decision‐Making Roles and Localizing Implementation Strategies

This study revealed marked differences in the perceptions and attitudes toward PtDAs between healthcare professionals and patients. Healthcare providers, particularly physicians, demonstrated greater familiarity with the concept of PtDAs; however, their willingness to use these tools was constrained by time pressures and insufficient operational skills [36]. In contrast, although patients exhibited limited awareness of PtDAs prior to exposure, the majority expressed a strong intention to use them after receiving explanations. These findings suggest that implementation challenges cannot be simply attributed to patient resistance. Instead, it is essential to enhance patient health education and foster awareness of participation in SDM [37].

Given the distinct demands and barriers among different interest holders, the implementation strategies for PtDAs must be tailored accordingly [14]. For physicians, strategies should address their specific concerns about efficiency and competence, perhaps by demonstrating how PtDAs can streamline the information‐sharing process and providing simplified, quick‐reference guides [32]. For nurses, implementation plans should explicitly integrate PtDA guidance into their scope of practice through policy clarification, targeted training, and support [38]. For patients, efforts must focus on normalizing their role in decision‐making through preconsultation materials and clear communication from all staff that their input is valued [39]. Within the unique context of China′s healthcare culture, clearly defining the roles of physicians, nurses, and patients, and establishing a complementary and collaborative decision‐making model are critical to the successful implementation of PtDAs [9].

### 4.4. Organizational and Cultural Imperatives

The barriers related to environmental context and resources, social influences, and behavioral regulation point to the necessity of an organizational‐level strategy. The lack of diverse PtDA formats (e.g., digital versions) limits accessibility, whereas the perceived lack of leadership support and a supportive organizational culture create a major impediment. Sustainable implementation requires more than just training frontline staff; it requires committed leadership, investment in appropriate resources, and the development of a clinic‐wide culture that genuinely values patient‐centered care and SDM [40, 41]. Incorporating the use of PtDAs into quality indicators or electronic health record systems could create the necessary “behavioral regulation” to encourage consistent use [13, 42].

### 4.5. Strengths and Trustworthiness

This study′s primary strength lies in its theory‐driven, multi‐interest‐holder design, which captures implementation determinants across the care continuum rather than isolating single interest holder perspectives. The application of the TDF ensures comprehensive mapping of behavioral and organizational determinants, enhancing analytical rigor and facilitating targeted strategy development. Trustworthiness was systematically maintained through prolonged engagement, methodological triangulation, reflexive documentation, peer audit, and explicit positionality management. Although conducted across a tertiary hospital and a community health center in Beijing, the thick contextual description and framework‐based findings offer transferable insights for other urban Chinese healthcare settings undergoing SDM integration. Future implementation trials across primary and secondary care tiers will further validate these determinants and refine context‐specific deployment strategies, ultimately assessing the impact of PtDA‐facilitated SDM on decision quality, patient satisfaction, and long‐term T2DM outcomes.

## 5. Conclusion

This TDF‐informed qualitative study characterizes the core determinants shaping PtDA implementation for insulin initiation decisions in Chinese patients with T2DM. Moving beyond conventional emphasis on consultation time, our findings reveal that foundational gaps in knowledge, practical facilitation skills, and structural role constraints represent the most immediate barriers to adoption. Conversely, patients′ willingness to engage in informed deliberation, nurses′ readiness to integrate decision support into existing education routines, and shared optimism regarding enhanced decisional clarity serve as critical catalysts. To translate these insights into sustainable practice, PtDAs should be embedded through a multilevel, context‐sensitive implementation strategy: (1) targeted competency training for both clinicians and patients to bridge knowledge and skill gaps; (2) explicit role delineation that formally positions nurses as facilitators of preference clarification within their scope of practice; (3) contextual adaptation of PtDA formats and delivery workflows to align with real‐world clinical demands and health literacy levels; and (4) sustained organizational commitment through leadership endorsement, performance integration, and continuous monitoring mechanisms. By addressing these theory‐mapped determinants, healthcare systems can leverage PtDAs not as instruments to drive insulin uptake, but as neutral, evidence‐based tools that foster high‐quality, preference‐congruent SDM, ultimately improving care alignment and long‐term T2DM management outcomes.

**Nomenclature**
HbA1c: glycated hemoglobin
PtDAs: patient decision aids
T2DM: Type 2 diabetes mellitus
TDF: Theoretical Domains Framework
SDM: shared decision‐making

## Author Contributions

Dan Yang: conceptualization, methodology, formal analysis, investigation, data curation, writing—original draft, and writing—review and editing. Hongzhan Jiang: conceptualization, methodology, formal analysis, investigation, data curation, writing—original draft, and writing—review and editing. Meiqi Meng: investigation, data curation, and writing—review and editing. Ziyan Wang: investigation, data curation, and writing—review and editing. Wanting Shen: investigation, data curation, and writing—review and editing. Hongxin Liu: resources, supervision, and writing—review and editing. Haiou Yang: resources, supervision, and writing—review and editing. Xuejing Li: conceptualization, resources, supervision, project administration, and writing—review and editing. Yufang Hao: conceptualization, resources, supervision, project administration, funding acquisition, and writing—review and editing. Dan Yang and Hongzhan Jiang contributed equally to this work.

## Funding

The Traditional Chinese Medicine Innovation Team and Talent Support Program—National Traditional Chinese Medicine Multidisciplinary Cross‐Innovation Team Project—provided financial assistance for this study, ZYYCXTD‐D‐202401.

## Disclosure

All authors have read and approved the final version of the manuscript. Yufang Hao had full access to all of the data in this study and takes complete responsibility for the integrity of the data and the accuracy of the data analysis.

## Ethics Statement

This study was conducted in accordance with the ethical principles of the declaration of Helsinki and was approved by the Institutional Review Board of Beijing University of Chinese Medicine (Approval Number: 2024BZYLL0709).

## Consent

Written informed consent was obtained from all participants prior to their inclusion in the study.

## Conflicts of Interest

The authors declare no conflicts of interest.

## Supporting Information

Additional supporting information can be found online in the Supporting Information section.

## Supporting information


**Supporting Information 1** Supporting Information S1: Patient decision aid tool for insulin initiation.

**Supporting Information 2** Supporting Information S2: Interview guide for barriers and facilitators of PtDA implementation.

**Supporting Information 3** Supporting Information S3: Illustrative verbatim quotes supporting thematic findings.

## Data Availability

The authors confirm that the data supporting the findings of this study are available within the article and/or its supporting information.
