# Supplementary material for: Implementing Patient Decision Aids for Insulin Initiation in China: What are the Barriers and Facilitators? A TDF‐Based Qualitative Study
Source: J Diabetes Res. 2026 Jul 8;2026:2842572. doi: 10.1155/jdr/2842572 (PMC13343304; doi:10.1155/jdr/2842572)
Supplement: Supplementary file 1 — Supporting Information 1 Supporting Information S1: Patient decision aid tool for insulin initiation. [file JDR-2026-2842572-s001.docx]

Supplementary Material S1

Patient Decision Aid Tool for Insulin Initiation


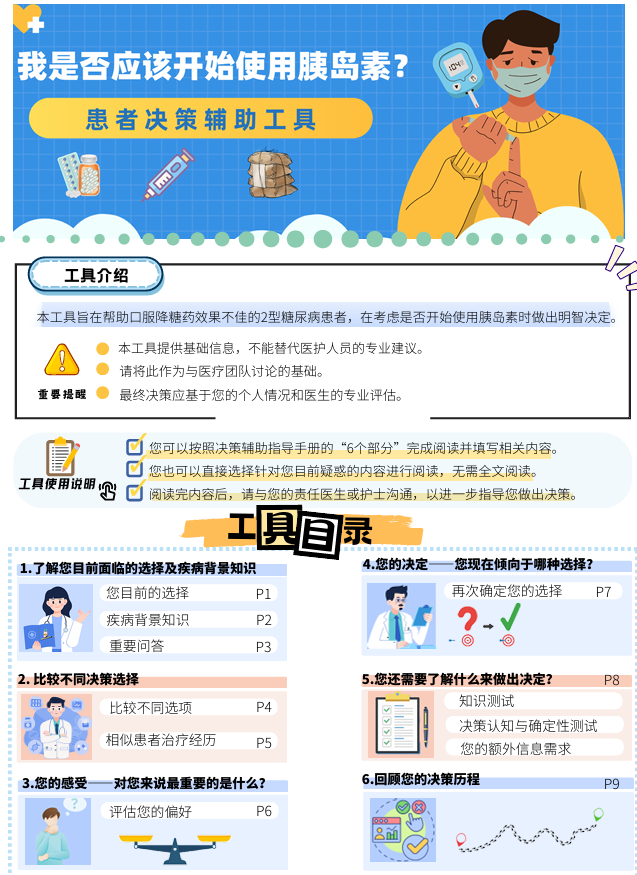


**Tool Catalogue**

- Understand your current treatment options and background knowledge of your condition
- Compare different decision options
- Your priorities – what matters most to you
- Your decision – which option do you lean toward now
- What additional information do you need to make a decision
- Review your decision-making journey

**Instructions for Use of the Tool**

- You may read and fill in relevant contents following the six sections specified in the decision aid guidebook.
- You may directly read the sections addressing your current doubts without going through the entire document.
- After reading, please consult your attending doctor or nurse for further guidance to help you make a decision.

# Tool Introduction

This tool is designed to help patients with type 2 diabetes who are considering options for improving glycemic control make informed decisions about insulin initiation.

- It provides general information only and shall not replace professional medical advice from healthcare providers.
- You may use this material as a reference for discussions with your medical team.
- The final decision should be made based on your individual health status and professional assessment by your doctor.

Should I start using insulin?

Patient Decision Aids


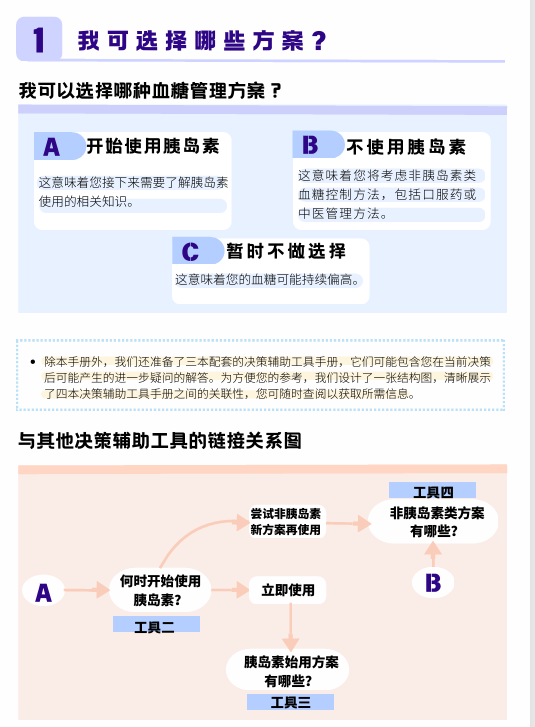


Try non-insulin regimens

Use Immediately

Tool 4: What Are the Non-Insulin Regimens?

Tool 3: What Are the Initial Insulin Regimens?

Tool 2: When to Start Insulin Therapy?

In addition to this handbook, we have prepared three supplementary decision aid tool handbooks, which may provide answers to further questions you may have after making your current decision. For your easy reference, we have created a structural diagram that clearly illustrates the correlations among the four decision aid tool handbooks, which you may review at any time to obtain the information you need.

Which blood glucose management plan can I choose?

A. Start insulin therapy – This means you will need to learn relevant knowledge about insulin administration.

B. Avoid insulin therapy – This means you will consider non-insulin blood glucose control methods, including oral medications and traditional Chinese medicine interventions.

C. Defer the decision temporarily – This means your blood glucose levels may remain elevated.
