# Supplementary material for: Implementing Patient Decision Aids for Insulin Initiation in China: What are the Barriers and Facilitators? A TDF‐Based Qualitative Study
Source: J Diabetes Res. 2026 Jul 8;2026:2842572. doi: 10.1155/jdr/2842572 (PMC13343304; doi:10.1155/jdr/2842572)
Supplement: Supplementary file 2 — Supporting Information 2 Supporting Information S2: Interview guide for barriers and facilitators of PtDA implementation. [file JDR-2026-2842572-s002.docx]

**Supplementary Material S2**

**Interview Guide for Barriers and Facilitators of PtDA Implementation**

| **TDF** | **Definition** | **Interview Outline-Patient** | **Interview Outline-Medical Staff** |
| --- | --- | --- | --- |
| knowledge | The understanding of things | 1. Have you been exposed to similar patient decision aids before (such as health apps, educational manuals)? What impact did it have on your management choices? Why?  2. Do you think PDAs can help you compare the advantages and disadvantages of insulin, non-insulin, and traditional Chinese medicine management plans? What specific information do you hope it will provide? Why? | 1. Are you familiar with patient decision aids? What aspects do you know about them?  2. How do you obtain the latest information and guidelines on the use of PDAs? |
| Memory, Attention & Decision Processes (Awareness) | An individual’s ability to retain information, selectively focus on various aspects of the environment, and make choices between two or more alternatives | 1.What designs of PDAs are most likely to catch your attention? (Such as structure, catalog, prompt information, red warning prompts)  2. When viewing relevant information provided by PDAs (such as Chinese and Western medicine treatments), what content is easy to make you find it difficult to understand? | 1. Which modules or contents in PDAs are most likely to make you forget or overlook them? Why?  2. In what scenarios would you be willing to use PDAs? Why? |
| Behavioural Regulation | Designed to manage or change objectively measured behaviors | 1. Based on the option information provided by PDAs, through which methods will you verify their reliability? (Such as consulting a doctor, referring to other materials)?  2. What methods do you think can help you better understand this tool? | 1. If you were to apply PDAs to your workflow, would you adjust the existing workflow to improve the use of PDAs? Why?  2. Based on your experience, what strategies can better help medical staff understand and implement this tool? |
| Skills | Designed to manage or change objectively measured behaviors | 1. If PDAs require you to input your own preference evaluations and some simple knowledge tests, can you complete these operations independently?  2. What operational obstacles (such as complex interfaces, difficult-to-understand terminology) might make you abandon the use of PDAs? Why? | 1. Do the content forms of existing PDAs support patients in better understanding different Chinese and Western medicine treatment plans? Why?  2. What skills do you think are needed to effectively guide patients in using PDAs? Why? |
| Environmental Context & Resources | Any environment or situation that an individual is in, which can hinder or encourage the development of their skills and abilities, independence, social skills, and adaptive behaviors | What factors in the hospital do you think would hinder or promote your use of this tool (such as a dedicated communication room, video materials, personal explanations, publicity boards, etc.)? | 1. Currently, do the information systems, WeChat groups, mini-programs, official accounts, etc. in your department support PDA data docking? Why?  2. What kind of resource shortages do you think will hinder the promotion of PDAs? Why? |
| Social Influences | The interpersonal communication process that can make individuals change their thoughts, feelings, or behaviors | Do you think doctors, nurses, or your family members will affect your use of this tool? | 1. How does the attitude of the department leaders towards PDAs affect your willingness to use them? Why?  2. Do the evaluations of PDAs by peers (such as doctors from other hospitals) affect your opinion? Why? |
| Social/Professional Role & Identity | A series of behaviors and personal qualities of an individual in a social or work environment | 1. What is your view on your role in the decision-making process for initiating insulin use? (For example, making the decision independently, mainly following the doctor's advice, discussing the decision with the doctor).  2. What impact do you think using this tool will have on your decision-making discussions with doctors? | 1. Who do you think should be responsible for the promotion and training of PDAs in the department/clinic, and why?  2. Does your workload allow you to provide PDA usage guidance to each patient? Why? |
| Intentions | Consciously making a behavioral decision or resolving to act in a certain way | Are you willing to find a management plan that suits you according to the guidance of PDAs? In what situations would you be less willing? Why? | 1. If possible, how do you plan to integrate PDAs into the existing diagnosis and treatment process? Why?  2. Are you willing to participate in the iterative optimization of PDAs? What suggestions do you have? |
| Beliefs about Capabilities | Recognize the authenticity, reality, or effectiveness of an individual's ability to actively apply their capabilities or talents | 1. Can the information in PDAs comparing the efficacy, side effects, etc., of insulin, traditional Chinese medicine interventions, and Western medical treatments help you understand their differences and the possibility of combined use?  2. How confident are you in your ability to use PDAs well? Why? | How confident are you in balancing different plans and patients' needs through PDAs? Why? |
| Optimism | Believe that things will develop in the best direction or achieve the expected goals | 1. Do you think using PDAs can reduce your hesitation when choosing insulin, non-insulin western medicine, or traditional Chinese medicine? Which functions are most likely to achieve this?  2. Do you expect PDAs to change the way you discuss treatment plans with doctors? Why? | To what extent do you expect PDAs to reduce patients' decision conflicts or decision hesitations? Why? |
| Beliefs about Consequences | Recognize the authenticity, reality, or validity of the behavioral results in a specific situation | What impact do you think using this tool will have on your decisions during the insulin initiation phase? Why? | 1. If PDAs are not accepted by patients for use, what do you think are the main possible reasons?  2. Is it possible that using PDAs will increase your workload? Why? |
| Goal | The mental representation of an individual’s desire to achieve a certain result or final state | 1.When deciding whether to start insulin therapy, what specific questions do you most hope PDAs will help you clarify? (For example, “Can traditional Chinese medicine delay the use of insulin?”)  2. Are these goals consistent with the doctor's expectations for your disease management? Why? | 1. What clinical goals do you think can be achieved through PDAs? Why?  2. Are these goals consistent with the expectations of the department management? Why? |
| Reinforcement | Increasing the probability of a response by arranging dependencies or contingencies between the response and a given stimulus | If using PDAs can help you reach a consensus with doctors faster (such as reducing the communication time during outpatient visits), would this increase your willingness to use them?  What incentive measures do you think could encourage you to use this tool? | 1. What incentive mechanisms do you think would motivate you to recommend PDAs more actively?  2. Do you think hospitals include the use of PDAs in their quality assessment indicators? |
| emotion | A complex response pattern that an individual exhibits when faced with important issues or events, which is a physiological and psychological state resulting from the integration of various complex feelings, thoughts, and behavioral manifestations. | 1. What functions of PDAs make you feel uneasy? (e.g., patient stories)  2. How do you hope PDAs can help you relieve choice anxiety? (e.g., instant online consultation, doctor-patient shared decision records) | 1. When using PDAs, in what situations might you feel frustrated? Why?  2. What kind of support do you think can alleviate the negative emotions during the use of PDAs? |
