# Supplementary material for: Implementing Patient Decision Aids for Insulin Initiation in China: What are the Barriers and Facilitators? A TDF‐Based Qualitative Study
Source: J Diabetes Res. 2026 Jul 8;2026:2842572. doi: 10.1155/jdr/2842572 (PMC13343304; doi:10.1155/jdr/2842572)
Supplement: Supplementary file 3 — Supporting Information 3 Supporting Information S3: Illustrative verbatim quotes supporting thematic findings. [file JDR-2026-2842572-s003.docx]

Supplementary Table S3

Illustrative Verbatim Quotes Supporting Thematic Findings

| **TDF Domain** | **Interest Holder Group** | **Barrier (※) / Facilitator**  **(★)** | **Illustrative Quote**  **(English translation)** |
| --- | --- | --- | --- |
| **Knowledge** | Patient | ※ Lack of Awareness and Knowledge Regarding PtDAs | “I had never heard of decision aids before. When I looked at it, I didn’t really understand how to use it or how it could help me.” (P1) |
|  |  |  | “I think the doctor is the expert; they should tell me what to do. I’m afraid this kind of tool might confuse me even more.” (P4) |
|  | Healthcare Professional | ※ Insufficient Knowledge of PtDA Content | “I have some understanding of decision aids, but I’m not very clear on how to use them. If I were to explain to patients using this tool, I might be uncertain about the sources of some data in the tool myself.” (D2) |
|  |  |  | “If I weren’t involved in creating this tool and didn’t know the details, I might have reservations about recommending it to patients.” (D3) |
| **Skills** | Patient | ※ Lack of skills to use PtDAs | “What do the numbers and percentages in that table mean?” (P2) |
|  |  |  | “I think the content is good, but how do I use it? Can I just make a choice after reading this? Is it reliable?” (p3) |
|  | Healthcare Professional | ※ Insufficient skills to guide PtDA implementation | “I’m worried that if I ask patients to use this tool and they encounter problems during use, I might not be able to guarantee I can answer them well.” (N3) |
|  |  | ★ Existing foundation in health education practices | “I usually explain injection methods, potential side effects, and precautions in detail before patients start insulin. These contents in the PtDA align well with my usual education, just in a more visual format.” (N3) |
|  |  |  | “We provide related education to patients before and after medication, and also explain some principles during treatment and care. This tool integrates these contents. If used as educational material, it could serve our health education work very well.” (N6) |
| **Social/Professional Role & Identity** | Patient | ★ Acceptance of patient’s role in decision-making | “Previously I thought this was entirely the doctor’s business. After your introduction to this tool, I feel I can also participate in discussing my current treatment plan with the doctor, as it’s my own body after all.” (P4) |
|  |  |  | “I think it’s true that treatment plans should be discussed with the doctor. This tool is very detailed and includes comparisons of different options, which helped me understand the differences between medications better.” (P7) |
|  | Healthcare Professional | ※ Physicians self-identify as “decision-makers” not “guides” | “To be honest, I still think doctors play a larger role in decision-making.” (D2) |
|  |  |  | “Considering most patients’ limited knowledge levels, doctors ultimately decide the medication choice in the vast majority of cases, and patients also trust doctors. A possible concern is that using PtDAs might increase the complexity of diagnosis and treatment.” (D6) |
|  |  | ※ Role ambiguity and scope-of-practice constraints | “We nurses are mainly responsible for executing medical orders and health education. Participating in patient treatment decisions might not be our primary duty.” (N3) |
|  |  |  | “Currently, we are mostly on the wards and don’t really participate in patient treatment decisions. Assisting patient decision-making is probably more on the doctor’s side.” (N5) |
|  |  | ★ Alignment with professional values and evolving role identity | “Using this tool could indeed save a lot of time spent explaining to p)atients and help us communicate better with them.” (D1) |
|  |  |  | “There’s a focus on patient-centeredness now, and I think guiding patient participation in decision-making is something we should do. PtDAs are a good guiding tool.” (D3) |
| **Beliefs about Capabilities** | Patient | ※ Lack of confidence in using PtDAs for decision-making | “My education level isn’t high. Although I can understand these contents, combining them to make up my own mind might be a bit difficult.” (P6) |
|  |  |  | “I think the tool’s content is very rich... But I don’t know how to communicate my choice with the doctor after reading it. I feel that even though I understand after reading, I still think it's best to follow the doctor’s advice.” (P7) |
|  | Healthcare Professional | ※ Lack of confidence in using PtDAs | “If using this tool requires further learning... it may be a bit difficult to adapt to.” (D4) |
|  |  |  | “Our daily work is already very busy, with not enough time to familiarize ourselves with this tool. If we can’t use it proficiently, it might instead affect patients’ trust in my professional ability.” (N5) |
|  |  | ★ Belief that PtDAs can enhance decision-guiding capabilities | “PtDAs provide standardized information and processes, which may allow me to explain the treatment plan options to patients during this period more comprehensively.” (D4) |
|  |  |  | “Judging from the current content and form of the PtDA, it’s a good choice tool for guiding patients planning to use insulin, adjust current medication regimens, or wanting to use TCM therapies.” (N7) |
| **Reinforcement** | Healthcare Professional | ※ Lack of performance evaluation and incentive mechanisms | “Currently, using PtDAs is still a non-routine process in diagnosis and treatment. There are no departmental supervision or additional requirements for use, so it’s unlikely to be widely adopted.” (D3) |
|  |  |  | “If there were clear incentive measures, like incorporating PtDA use into evaluations or granting certain workload recognition, I believe everyone would be more active in adopting this tool.” (N6) |
| **Intentions** | Patient | ★ Positive intention to use PtDAs during medical consultations | “I think it would be great if such a tool were available during my medical visits.” (P1) |
|  |  |  | “I am very willing to use this tool to learn more about my condition and options.” (P3) |
|  | Healthcare Professional | ※ Lack of motivation to provide decision support following PtDAs | “The tool’s content is very rich... how to guide patients in using it is my current confusion... Considering outpatient time constraints, it might be difficult to implement.” (D3) |
|  |  |  | “I feel my current workload is already heavy, and I might not have the energy to learn how to use this tool effectively.” (N1) |
| **Beliefs about Consequences** | Patient | ★ Belief in enhanced understanding and decisional clarity | “After viewing this tool, I felt I understood things more clearly. It answered my confusing questions, like the differences between insulin and other medications, and differences between other oral medications.” (P3) |
|  |  |  | “This tool showed me the advantages and disadvantages of different options... I would choose a plan more suitable for my lifestyle habits, making me more willing to adhere to treatment.” (P7) |
|  | Healthcare Professional | ★ Recognition of PtDAs’ positive impact on clinical decision-making | “Using this tool, I believe patients will have fewer questions and won’t need repeated explanations during visits. Although initial use might take more time, in the long run, it should actually save our workload.” (D6) |
|  |  |  | “If patients use this tool before their visit, it should reduce many of their doubts, especially regarding different medications and their concerns about side effects.” (N4) |
| **Optimism** | Patient | ★ Positive attitude towards PtDAs for assisted decision-making | “I think this kind of tool is really good. It answered many of my questions, and I can scan the QR code to watch related videos... which gives me more confidence for subsequent treatment.” (P2) |
|  |  |  | “This tool was relatively easy for me to understand, especially the comparison of different options... communicating with the doctor will be easier.” (P4) |
|  | Healthcare Professional | ★ Optimism about gradual clinical implementation of PtDAs | “I am optimistic about the prospects of this tool. Although it might increase workload initially, in the long term, it can help us explain different options more systematically, reduce time spent on repetitive explanations, and improve communication efficiency.” (D5) |
|  |  |  | “This tool isn’t only for patients currently facing possible insulin use; early-stage diabetes patients can also view it, especially related disease knowledge and medication aspects.” (N6) |
|  |  | ※ Concern that PtDAs may increase decision complexity | “I’m concerned that some patients, especially the elderly or those with lower education levels, might feel confused by the large amount of information... they might become indecisive, which could delay treatment.” (D5) |
|  |  |  | “The worry is encountering more stubborn patients who interpret the tool’s information too extremely. For example, if they need immediate insulin but, after viewing the tool, one-sidedly think they can just use TCM or oral Western medicine instead, delaying their condition.” (N4) |
| **Environmental Context & Resources** | Patient | ※ Limited PtDA formats, lack of diverse presentation methods | “I think it would be even better if there were a video explanation version.” (P3) |
|  |  |  | “I think it would be better if the content on this could be viewed on a mobile phone.” (P5) |
|  | Healthcare Professional | ※ Inadequate PtDA support resources, lack of diverse formats | “Our patients cover a wide age range... Younger people might prefer electronic versions or WeChat mini-programs, while older people might still prefer paper materials.” (D4) |
|  |  |  | “I think patients would find it easier to accept if there were video explanation formats.” (N5) |
|  |  | ※ Discrepancy between PtDA options and actual clinical options | “Some of the medication options listed in this PtDA are not entirely feasible in our hospital... If patients learn about these options... and then request these medications that are difficult to provide, it could create communication difficulties.” (D6) |
|  |  |  | “The specific option details... might not fully align with our hospital's practice. It would be more appropriate to adjust the content based on specific clinical situations before giving it to patients.” (N4) |
|  |  | ※ Human resource constraints and increased workload | “When outpatient volume is high, consultation time per patient is very limited. If we need to explain this tool in detail and answer various patient questions... it will definitely lengthen consultation time.” (D3) |
|  |  |  | “If the outpatient clinic is very busy, I don’t know how to efficiently guide patients in using PtDAs.” (D5) |
| **Memory, Attention & Decision Processes** | Patient | ※ Habituated to doctor-led decisions, lack of participation awareness | “Although this tool taught me a lot, I still think I’d better listen to the doctor, since the doctor is the expert.” (P4) |
|  |  |  | “I trust the doctor’s professional judgment; they know better than I do what treatment is best for me.” (P10) |
|  |  | ★ Willingness to actively participate, seeking informational autonomy | “I feel I have the right to know all possible options and the advantages and disadvantages of each. This tool provides the information I want, allowing me to better communicate my treatment plan with the doctor.” (P2) |
|  |  |  | “I think I should discuss the treatment plan more with the doctor. This tool’s content answered many questions I probably should have asked the doctor. I think it helps me further find the treatment that might be most suitable for me.” (P7) |
|  | Healthcare Professional | ※ Lack of habit in integrating PtDAs into clinical decision processes | “To be honest, each patient’s consultation time might only be 5 to 10 minutes. Suddenly changing this approach, taking extra time to use decision aids, really requires an adaptation process.” (D6) |
|  |  |  | “If ward nurses adjust the health education part to using this tool, I think it’s feasible. But if we need to additionally arrange to guide patients in using this tool for treatment or nursing decisions, it's a bit difficult.” (N5) |
| **Social Influences** | Patient | ※ Low awareness of PtDAs, lack of public promotion | “This is the first time I’ve heard of this kind of decision aid tool... I initially thought it was some kind of advertisement or promotion.” (P6) |
|  |  |  | “Seeing it for the first time, I didn’t quite understand why the doctor gave me this tool. Does it mean he himself isn’t sure how to treat me?” (P5) |
|  | Healthcare Professional | ※ Insufficient organizational culture | “To truly promote this tool, a cultural shift across the entire hospital is needed... it requires long-term awareness cultivation and system building.” (D2) |
|  |  |  | “We might need to gradually let more doctors and nurses understand the cultural background and significance of patient decision aids and integrate them into the consultation environment.” (N5) |
|  |  | ※ Limited leadership support | “Currently, hospital leadership hasn’t emphasized doctor-patient shared decision-making or encouraged the use of decision aids. Under such circumstances... I might use this tool selectively.” (D4) |
|  |  |  | “If hospital leadership valued the use of this decision aid tool, there should be specialized training, assessment, and reward mechanisms, not simply requiring us to add this task on top of our existing workload.” (N8) |
| **Behavioural Regulation** | Healthcare Professional | ※ Lack of monitoring mechanisms for decision-making behaviours | “Everyone might be quite active initially, but slowly, perhaps no one pays attention anymore. Because nobody checks or evaluates whether we are using it or how well we are using it.” (N3) |
|  |  |  | “Using PtDAs requires changing our habitual communication styles and decision processes. Such change isn’t easy.” (D3) |

PtDA = Patient Decision Aid; TCM = Traditional Chinese Medicine;
